# A clinico-anatomical dissection of the magnocellular and parvocellular pathways in a patient with the Riddoch syndrome

**DOI:** 10.1007/s00429-024-02774-8

**Published:** 2024-03-16

**Authors:** Ahmad Beyh, Samuel E. Rasche, Alexander Leff, Dominic ffytche, Semir Zeki

**Affiliations:** 1 Laboratory of Neurobiology, University College London, London, UK; 2 UCL Queen Square Institute of Neurology, University College London, London, UK; 3 Department of Old Age Psychiatry, Institute of Psychiatry, Psychology and Neuroscience, King’s College London, London, UK

**Keywords:** Statokinetic dissociation, Blindsight, V5/MT +, Magnocellular, Parvocellular, Optic radiations

## Abstract

**Key message:**

The Riddoch syndrome is thought to be caused by damage to the primary visual cortex (V1), usually following a vascular event. This study shows that damage to the anatomical input to V1, i.e., the optic radiations, can result in selective visual deficits that mimic the Riddoch syndrome. The results also highlight the differential susceptibility of the magnocellular and parvocellular visual systems to injury. Overall, this study offers new insights that will improve our understanding of the impact of brain injury and neurosurgery on the visual pathways.

**Abstract:**

The Riddoch syndrome, characterised by the ability to perceive, consciously, moving visual stimuli but not static ones, has been associated with lesions of primary visual cortex (V1). We present here the case of patient YL who, after a tumour resection surgery that spared his V1, nevertheless showed symptoms of the Riddoch syndrome. Based on our testing, we postulated that the magnocellular (M) and parvocellular (P) inputs to his V1 may be differentially affected. In a first experiment, YL was presented with static and moving checkerboards in his blind field while undergoing multimodal magnetic resonance imaging (MRI), including structural, functional, and diffusion, acquired at 3 T. In a second experiment, we assessed YL’s neural responses to M and P visual stimuli using psychophysics and high-resolution fMRI acquired at 7 T. YL’s optic radiations were partially damaged but not severed. We found extensive activity in his visual cortex for moving, but not static, visual stimuli, while our psychophysical tests revealed that only low-spatial frequency moving checkerboards were perceived. High-resolution fMRI revealed strong responses in YL's V1 to M stimuli and very weak ones to P stimuli, indicating a functional P lesion affecting V1. In addition, YL frequently reported seeing moving stimuli and discriminating their direction of motion in the absence of visual stimulation, suggesting that he was experiencing visual hallucinations. Overall, this study highlights the possibility of a selective loss of P inputs to V1 resulting in the Riddoch syndrome and in hallucinations of visual motion.

**Supplementary Information:**

The online version contains supplementary material available at 10.1007/s00429-024-02774-8.

## Introduction

George Riddoch’s description (1917) of the capacity of patients blinded by damage to their primary visual cortex to perceive moving visual stimuli consciously was a remarkable observation. Despite being initially dismissed by his peers, most notably Gordon Holmes (1918), and lying dormant for decades, it opened a veritable Pandora’s box of interesting observations not only about the anatomico-physiological basis of the syndrome but also about the relationship of cortical damage to conscious experience. In the work reported here, we describe results that raise, on the one hand, questions that revolve around the extent to which the syndrome is determined by damage to the input to V1 rather than processing in it and, on the other, the conscious dimension surrounding the visual perception of motion in such patients.

Studies of Riddoch syndrome patients using brain imaging techniques have shown that the cortical motion area V5 is activated when visual motion is perceived in their blind field (Ajina et al. 2015a, b; Arcaro et al. 2019; Beyh et al. 2023; Zeki and ffytche 1998). In the macaque brain, V5 receives direct input from the lateral geniculate nucleus (LGN) and the pulvinar of the thalamus (Benevento and Rezak 1976; Cragg 1969; Fries 1981; Sincich et al. 2004; Yukie and Iwai 1981) and, in humans, signals from very fast moving visual stimuli can reach V5 up to 40 ms before reaching V1 (Beckers and Zeki 1995; ffytche et al. 1995), indicating that the inputs to the two visual areas are anatomically, functionally, and temporally segregated. Experimental ablation studies in the macaque (Schmid et al. 2010) and tractography studies in humans (Ajina et al. 2015a, b) have shown that direct input to V5 from the LGN is the anatomical substrate underlying residual motion perception after V1 damage. Thus, V5 can support a crude and impoverished, but conscious visual motion perception in the absence of V1.

We describe the case of patient YL (not his real initials) who has a dense right homonymous hemianopia but is able to perceive visual motion in his blind field. Therefore, YL’s psychophysical profile matches that of the Riddoch syndrome; however, he has no direct injury to V1, which is instead partially deafferented. This raises puzzling questions, of how such a lesion can selectively destroy a patient’s ability to see static objects but spare their sensitivity to visual motion, and what cortical mechanisms might be involved in producing those selective deficits. By presenting YL with stimuli that recruit mainly the magnocellular (M) or the parvocellular (P) visual pathways (Kaplan 2014), we have determined that the P input to his V1 is selectively affected. We also observed that YL has a strong tendency to report perceiving visual motion during the experiment even in the absence of visual stimulation (hallucination of motion).

## Methods

### Patient

YL is a right-handed male in his late twenties. He underwent an operation for a left hemispheric, low-grade intraventricular tumour and developed a dense right homonymous hemianopia after surgery, three years prior to this study. Subsequent clinical testing revealed signs of residual visual motion perception in his blind (right) hemifield. He was referred to our study via a specialist outpatient visual service run at the National Hospital for Neurology and Neurosurgery in London. He gave informed written consent to participate in our study, which had been approved by the Yorkshire and the Humber—South Yorkshire Research Ethics Committee (NHS Health Research Authority) and UCLH/UCL Joint Research Office (protocol number 137605).

### Experiment 1

The aim of this experiment was to establish whether YL could consciously perceive visual motion in his blind field, and to determine his neural responses while doing so. We assessed the former with psychophysics and the latter with fMRI.

#### Psychophysical testing

We used achromatic random checkerboards (40% contrast) that were either static or drifted upward or downward at a speed of 20°/s (Fig. S1). The stimuli subtended 12° in width and 22° in height and were confined to YL’s blind (right) field, 6° to the right of the vertical meridian. During the psychophysics session, YL was asked to indicate the motion direction of the stimulus after each presentation, following a two alternative forced choice (2AFC) approach, and to indicate his certainty of the response on a three-point scale, one indicating “complete guess”, two “I think I saw motion, but I’m not sure of its direction”, and three “I definitely saw the stimulus moving up (or down)”.

#### MRI data acquisition and procedure

Based on the results of the psychophysics studies, YL underwent MRI scanning at the Wellcome Centre for Human Neuroimaging. We collected data on a 3 T Siemens Magnetom Prisma scanner (Siemens Healthcare GmbH, Erlangen, Germany) including structural, volumetric T1w images to assess the extent of his lesion, fMRI data to assess his neural responses to visual motion, and multishell diffusion MRI to perform a tractographic reconstruction of his optic radiations.

Structural imaging was based on the 3D magnetisation-prepared accelerated gradient echo (MPRAGE) sequence: repetition time (TR) = 2.53 ms; echo time (TE) = 3.34 ms; flip angle = 7°; matrix of 256 × 256; field of view = 256 mm; voxel size = 1 × 1 × 1 mm^3^.

To assess visual motion responses, we collected data from two fMRI runs during which we presented YL with the same random checkerboard stimulus (Fig. S1), either statically or in motion (20°/s), as well as a ‘blank’ condition during which no stimulus was shown. Each of the three conditions was presented eight times in blocks of approximately 20 s. To ensure that YL was fixating the screen’s centre, he engaged in a fixation task by pressing a button in response to a brief (300 ms) colour change in the fixation cross that occurred at random throughout the acquisition.

fMRI data was based on the blood oxygen level dependent (BOLD) signal, measured with a 2D T2*-weighted echo planar imaging (EPI) sequence: volume TR = 3360 ms; TE = 30 ms; flip angle = 90°; ascending acquisition; matrix of 64 × 64; voxel size = 3 × 3 × 3 mm^3^; 48 slices. Two fMRI runs were acquired. Field mapping images were also acquired using a dual-echo gradient echo sequence to assist with susceptibility distortion correction.

Diffusion MRI data was based on a 2D spin-echo EPI sequence: TR = 3500 ms; TE = 61 ms; flip angle = 88°; matrix of 110 × 110; voxel size = 2 × 2 × 2 mm^3^; 72 slices; multiband factor of 2; in-plane acceleration factor of 2. Images were acquired with four diffusion shells: 30 diffusion directions at b = 500 s∙mm^−2^, 60 directions at b = 1500 s∙mm^−2^, 90 directions at b = 2500 s∙mm^−2^, and 120 directions at b = 6000 s∙mm^−2^. In addition, 25 b = 0 s∙mm^−2^ were interleaved throughout the acquisition, and seven b = 0 s∙mm^−2^ volumes were acquired with the reverse phase encoding polarity to correct for susceptibility distortions.

#### MRI data pre-processing and analysis

The T1w image was skull-stripped using *optiBET* (Lutkenhoff et al. 2014), bias field corrected using the *N4* tool (Tustison et al. 2010), and rigidly aligned, using *flirt* (Jenkinson et al. 2002), to the 1 mm MNI T1w brain template as a substitute for AC-PC alignment. This aligned image served as the anatomical reference for subsequent pre-processing and analysis steps.

The first four volumes of each fMRI run were discarded to allow the scanner to reach steady state. The remaining images were corrected for motion and slice-timing differences using *SPM12* (http://www.fil.ion.ucl.ac.uk/spm/software/). The corrected images were then simultaneously corrected for geometric distortions (based on the acquired field map) and aligned to the T1w image using FSL’s *epireg* tool (Greve and Fischl 2009; Jenkinson et al. 2002), while maintaining the voxel size at 3 × 3 × 3 mm^3^. The BOLD time series images were then spatially smoothed with a Gaussian kernel of a full width at half maximum (FWHM) of 4.5 mm. This produced the final fMRI time series images that were used in subsequent analyses.

A standard GLM was fit to the time series, with a task effect (stimulus presentation) for each of the moving and static conditions, and six motion correction parameters as nuisance regressors. Categorical comparisons were performed to identify the brain regions in which activity increased in response to the presentation of the moving and static random checkerboards. All resulting statistical images were thresholded at a voxelwise significance level of *p* < 0.001. This was done in *SPM12*.

Raw DWI data was first corrected for noise and Gibbs ringing artefacts (Kellner et al. 2016; Veraart et al. 2016). A magnetic susceptibility field was then calculated using *topup* (Andersson et al. 2003) based on b = 0 s∙mm^−2^ images acquired with opposite phase encoding. All images were subsequently corrected for motion and eddy current distortions using *eddy* (Andersson and Sotiropoulos 2016) with outlier (signal dropout) slice replacement (Andersson et al. 2016), incorporating the *topup* field into this step. The anisotropic power map was derived from the pre-processed data using *StarTrack* (www.mr-startrack.com) and used to calculate a rigid affine transformation (six degrees of freedom) to the T1w image with *flirt* (Jenkinson et al. 2002). The rigid transformation was then applied to the diffusion data (kept at a 2 mm voxel size) with a spline interpolation to produce the final set of pre-processed images. The diffusion gradients were also rotated at this stage using the same transformation matrix.

The diffusion data was used to reconstruct the optic radiations connecting YL’s lateral geniculate nucleus (LGN) to his visual cortex. The data from the two highest shells (*b* = 2500 and 6000 s∙mm^−2^) were modelled with spherical deconvolution based on the damped Richardson-Lucy algorithm (Dell’Acqua et al. 2010, 2013) in StarTrack, according to the following parameters: fibre response *α* = 1.5; number of iterations = 200; amplitude threshold *η* = 0.001; geometric regularisation *ν* = 16. A probabilistic dispersion tractography approach was followed to explore the full profile of the fibre orientation distribution function (fODF) in each voxel according to the following parameters: minimum HMOA threshold = 0.001; number of seeds per voxel = 2000; maximum angle threshold = 45°; minimum fibre length = 35 mm; maximum fibre length = 200 mm. This was done using a manually defined seed region of interest in the LGN. The resulting tractogram was imported into TrackVis (http://trackvis.org/) where manual cleaning was performed and streamlines terminating in visual cortex were selected. The final dissected white matter tracts were divided into 100 equidistant segments and microstructural mean and standard deviation metrics were calculated for each.

### Experiment 2

The first experiment revealed that YL can perceive motion in his blind field consciously, that his visual cortex is responsive to moving but not static visual stimuli, and that his optic radiations, though damaged, still connect his visual cortex with the thalamus. This led us to hypothesise that the M and P systems are differentially affected in his brain; we therefore conducted additional experiments to address this question.

#### Psychophysical testing

We used achromatic sine wave checkerboards (Fig. S2) that varied in spatial frequency (0.3 or 1.4 cycles/°), contrast (20% or 80%), and speed (1 or 8°/s). We collected a total of 224 trials over seven task runs, which included 28 trials per condition. YL was asked to give his response and his certainty to the perceived direction of motion as in Experiment 1. The stimuli were confined to the same location in his blind field.

#### MRI data acquisition and procedure

Based on the psychophysics results, YL underwent further MRI scanning at the Wellcome Centre for Human Neuroimaging; we collected data on a 7 T Siemens Magnetom Terra scanner including structural, volumetric T1w images, and fMRI data.

A T1w volume was acquired based on a 3D fast low-angle shot (FLASH) sequence with the following parameters: TR = 19.5 ms; TE = 2.3 ms; flip angle = 24°; field of view = 364 × 426 × 288 mm^3^; voxel size = 0.6 × 0.6 × 0.6 mm^3^.

We collected two fMRI runs during which we presented YL with P- and M-type stimuli, as well as blank trials. The P stimulus was a sine wave checkerboard with a spatial frequency of 1.4 cycles/°, 90% contrast, and drifting at a speed of 1.5°/s (Figure S3). The M stimulus had a spatial frequency of 0.35 cycles/°, 30% contrast, and a speed of 16°/s (Fig. S3). Each stimulus was presented eight times in blocks of approximately 24 s, interleaved by blank blocks of approximately 12 s. The stimulus subtended 20° in width and 10° in height due to the limited screen size at 7 T, simultaneously targeting both hemifields, and was masked with a grey disk (3° in diameter) in the centre to ensure that the fixation cross remained visible. Here, again, YL engaged in a fixation task.

The BOLD signal was measured with a 3D T2*-weighted EPI sequence: volume acquisition time = 2332 ms; TR = 53 ms; TE = 20 ms; flip angle = 15°; field of view = 192 × 192 × 88 mm^3^; voxel size = 1 × 1 × 1 mm^3^; PAT acceleration factor of 8; partial Fourier 6/8 in the phase-encoding direction. Four additional EPI volumes were acquired with the opposite phase encoding to be used later for distortion correction.

The main differences between psychophysics and fMRI in the second experiment were (1) the speed of the M stimulus and (2) the stimulated field of view. Regarding the first point, we chose a higher speed for the M stimulus during fMRI to ensure that BOLD signal changes to motion stimuli were strong enough and distinguishable enough from the slow stimulus. As for the second point, we only stimulated the blind field in psychophysics to ensure that the patient was not aware of the stimulus properties (e.g., texture or contents) during the direction discrimination task, while during fMRI we stimulated both hemifields simultaneously to include as many blocks of the localiser task as possible after having established the behavioural responses offline.

#### MRI data pre-processing and analysis

The T1w image was aligned with the structural image from the 3 T session for ease of comparison and served as the reference for the 7 T fMRI pre-processing steps. This was achieved through a rigid-body alignment performed using *flirt* (Jenkinson et al. 2002).

The fMRI images were first denoised using NORDIC (Vizioli et al. 2021). Then, the first two volumes of the first run were combined with their opposite phase-encoding counterparts and passed to *topup* to calculate the susceptibility distortion field (Andersson et al. 2003). Afterwards, the images from both fMRI runs were concatenated and passed to *eddy* (Andersson and Sotiropoulos 2016) where motion correction and susceptibility distortion correction (based on the *topup* field) were simultaneously applied, accounting for the effect of motion on these distortions (Andersson et al. 2018). The corrected images from both task runs, all of which were in alignment at this stage, were then aligned to the structural T1w image by way of a rigid-body alignment performed in *flirt* (Jenkinson et al. 2002) using the mutual information cost function and spline interpolation. Finally, the images were spatially smoothed with a Gaussian kernel of a FWHM of 1.0 mm.

The first four volumes of the time series were discarded, and a standard GLM was fit to the data with a task effect (stimulus presentation) for each of the M- and P-type conditions, and six motion correction parameters as nuisance regressors. Categorical comparisons (t test) were performed to identify the brain regions in which activity increased in response to the presentation of each type of stimulus relative to a grey background; additionally, the two conditions were directly compared with each other. All resulting statistical images were thresholded at a voxelwise significance level of *p* < 0.001. This was done in *SPM12*.

### Statistical analysis

#### Performance

We calculated YL’s accuracy on the motion direction discrimination task as the percentage of correct trials. For instance, for a given condition (e.g., low frequency, high speed, high contrast), accuracy, *A*, was calculated as:

1 $$A=\frac{{N}_{correct}}{{N}_{correct}+{N}_{incorrect}}\times 100$$

Given that the direction response had two possible outcomes only with equal probability (50% each), we used the binomial distribution based on the appropriate number of responses (trials) to determine whether accuracy was significantly above chance for a given task condition. We performed these analyses using the *binocdf* function in MATLAB (The MathWorks).

#### Certainty

To ease the interpretation of the certainty scores, which were collected on a three-point scale, we converted each trial’s certainty score to a percentage value as follows:

2 $${C}_{perc}=\frac{C-1}{2}\times 100$$

where *C* is the original score obtained on the three-point scale (values between 1 and 3), and *C*_*perc*_ is the certainty score in percentage terms. Therefore, *C*_*perc*_ = 0% would indicate the lowest certainty possible (i.e., complete guess), *C*_*perc*_ = 50% would indicate moderate certainty, and *C*_*perc*_ = 100% would indicate the highest level of certainty.

#### Certainty vs. accuracy

To assess whether YL’s certainty for each condition was related to his accuracy, we used Pearson’s correlation with one-tailed significance testing. We did this to test the direct relationship between mean certainty and accuracy for the eight conditions, and to test the correlation between the observed data and the data predicted by the psychophysical model proposed by Zeki and ffytche (1998).

#### Effect of stimulus properties

We further tested YL’s responses to assess the effect of spatial frequency, contrast, and speed on his performance. We trained a linear model, using maximum likelihood estimation, on 75% of the non-blank trials (*N* = 158) to predict whether YL’s response to a given combination of spatial frequency, contrast, and speed would be correct using frequency as the only predictor (Model 1). We then trained a second model using all three factors as predictors (Model 2). We then applied the same approach and trained two linear models to predict YL’s certainty using frequency as a sole predictor (Model 3) or using all three factors as predictors (Model 4). We tested each model’s predictions on the remaining 25% of trials (*N* = 53) to assess whether it generalises well to the rest of YL’s responses. We also applied the likelihood ratio test to assess whether including speed and contrast improved each model’s predictive power.

## Results

### Experiment 1

**Fig. 1:**
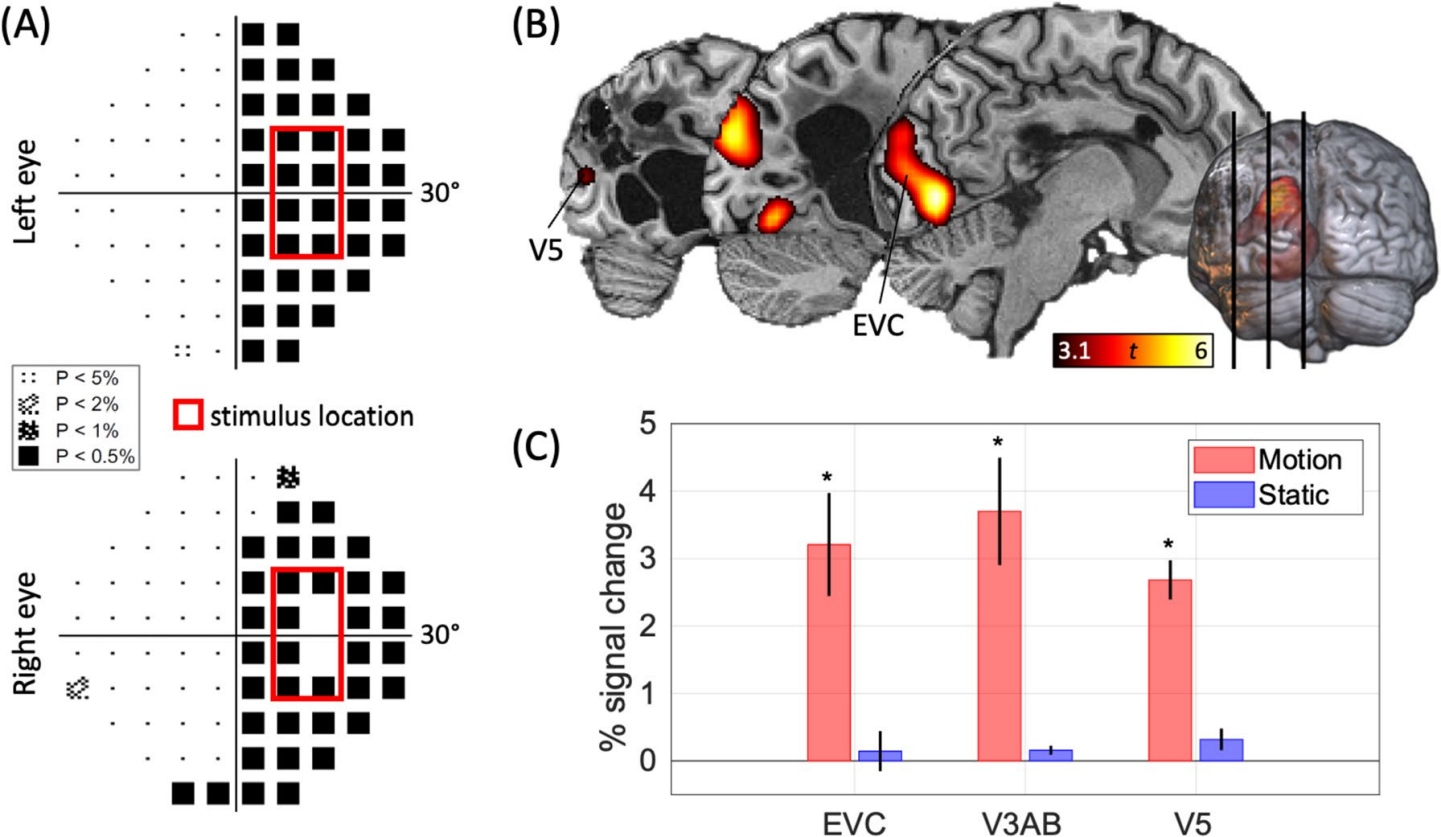
Strong responses to visual motion in the blind field in YL’s brain driven by direct thalamic input. **A** Perimetry results revealed that YL has a dense homonymous right hemianopia. The plots shown here are statistical displays that correspond to pattern deviation, i.e., the percentage of the normal population who measure below the patient’s value at each retinal point, corrected for optical impairments that affect the eye. The black squares indicate that YL is unable to detect bright flashes of light presented in his right visual field, while the small dots show that his vision is normal in the left visual field. The red contours indicate the location of the stimulus used during psychophysical and fMRI testing. **B** fMRI activity in left visual cortex in response to fast-moving random checkerboards (Fig. S1) presented in YL’s blind field (*p* < .001). **C** fMRI BOLD signal changes in left early visual cortex (EVC), V3A and V3B, and V5 were strong in response to drifting random checkerboards, but absent when the same checkerboards were static

Static Humphrey perimetry (30–2) revealed that YL had a dense homonymous right hemianopia (Fig. 1A). During psychophysical testing, he was very accurate in discriminating the direction of motion of drifting random checkerboards presented in his blind field (81% accuracy, *p* < 0.001) and was conscious of having seen them (71% certainty).

**Fig. 2:**
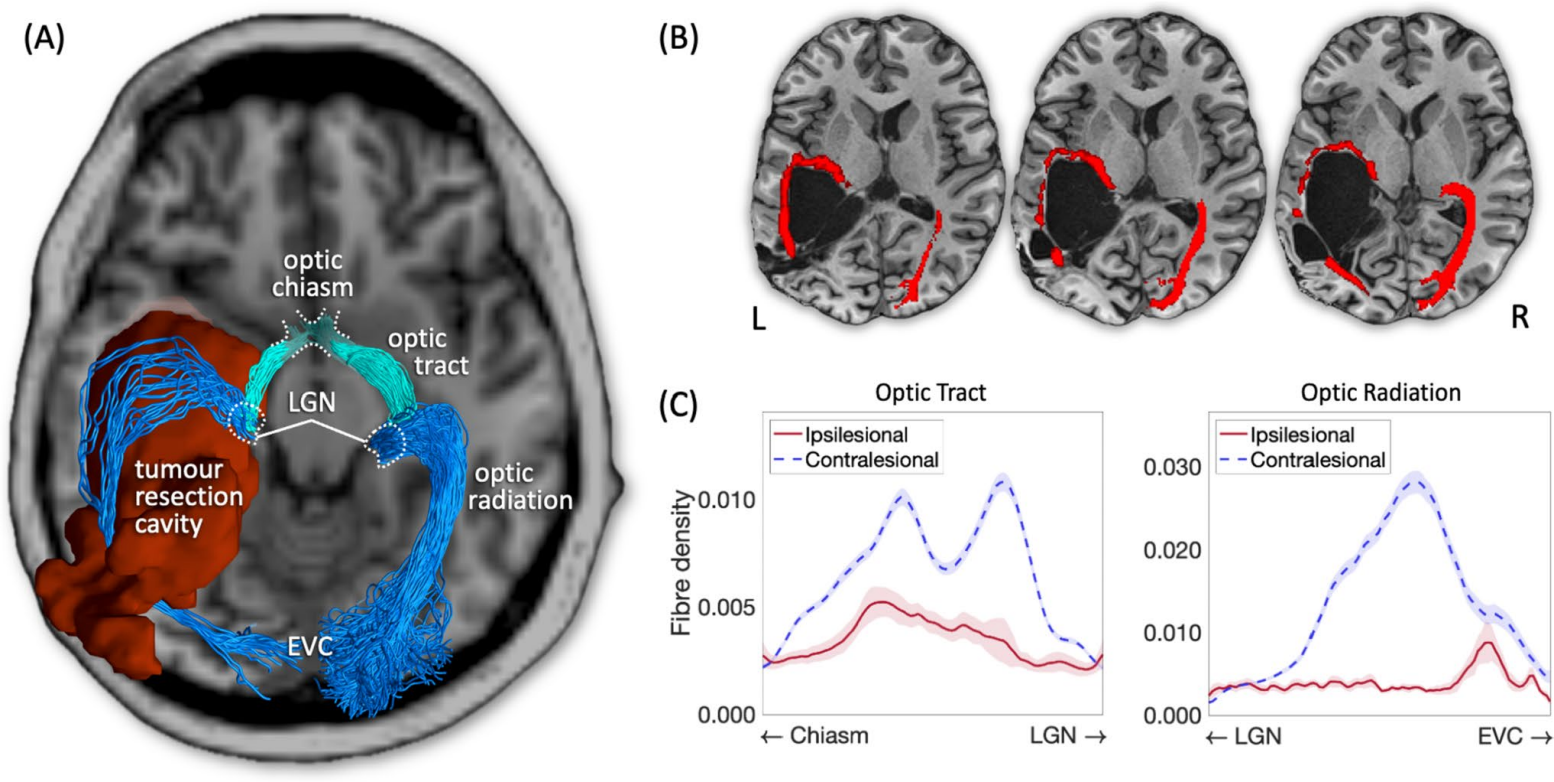
Tractographic reconstruction of the main visual pathways in patient YL. **A** 3D visualisation of the optic tract, optic radiation, and tumour resection cavity in YL’s brain. The optic radiation in the left hemisphere is clearly stretched and thin compared with the right hemisphere, and the left optic tract has a visibly smaller volume compared with the right one. **B** Axial slices showing the trajectory of the ipsilesional optic radiation in the vicinity of the tumour resection cavity. Note the very narrow spared white matter tissue where the optic radiation can pass into the occipital lobe. **C** Microstructural assessment of the visualised white matter tracts. The ipsilesional tracts are clearly severely affected in the aftermath of the surgery, which compromises their ability to transfer visual signals effectively. Note that the ipsilesional optic tract, which was not directly affected by the surgery, is likely showing signs of Wallerian degeneration

Prior to surgery, the intraventricular tumour had substantially expanded, compressing the white matter of YL's left temporal lobe, and displacing subcortical structures such as the thalamus and basal ganglia. Histological diagnosis after the resection indicated that the tumour was a low-grade glioma with the methylation class of a supratentorial subependymoma. Structural imaging revealed a large post-operative resection cavity with thin and degraded white matter tissue in the temporal lobe, but with sparing of the grey matter. The lesion extended posteriorly into the temporo-parietal junction and medially into the inferior parietal lobe, involving the posterior horn of the lateral ventricle. The occipital lobe was spared. Despite this, using tractography we were able to track white matter connections between the thalamus and visual cortex (Fig. 2).

fMRI revealed that YL’s visual cortex in general is highly responsive to moving random checkerboards (Fig. 1B,C). The BOLD signal change associated with these stimuli was highly significant in a large portion of visual cortex, spanning medial, dorsal, and lateral visual cortical areas, including early visual cortex (EVC; V1, V2, V3), V3A/B, and V5. In contrast, the same checkerboards failed to elicit any significant activations when they were static.

### Experiment 2

In the first experiment, there was a very strong response in EVC, including visual areas V2 and V3, to moving stimuli but an insignificant response to their static counterparts, despite a direct subcortical input to visual cortex in general (Benevento and Rezak 1976; Cragg 1969). This led us to hypothesise that the M and P visual pathways may have been affected differentially by the lesion. It was also difficult, due to the limited resolution of the 3 T fMRI data, to ascertain whether V1 in and around the calcarine sulcus was active, or whether we were instead measuring the partially overlapping signal from neighbouring V2. Therefore, we extended our studies by using high-resolution fMRI at 7 T to determine whether the P and M inputs had been differentially compromised.

**Fig. 3:**
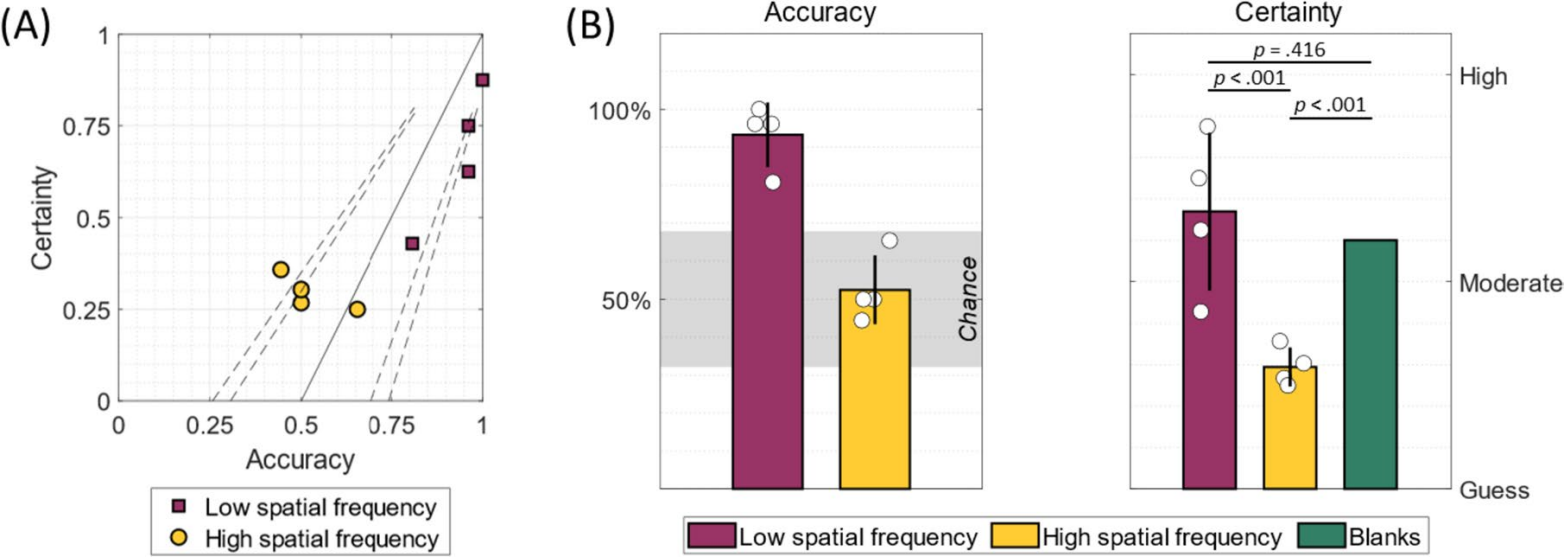
Different responses to spatial frequency. **A** YL’s accuracy and certainty per condition during the visual motion task where he had to discriminate the direction of motion of a stimulus presented in his blind field (see Table S1 for the scores per condition). YL’s performance was highly influenced by the spatial frequency of the stimulus. The solid line represents a psychophysical model adopted from Zeki and ffytche (1998) that assumes that certainty and accuracy are strongly linked; the dashed lines represent the boundaries of the model under the binomial distribution at *p* < .05 and *p* < .01, calculated for 28 trials per condition. YL’s psychophysical profile largely resembles the model’s prediction (*r* = 0.88, *p* = .002). **B** The same data from the first plot grouped by spatial frequency, with the addition of the mean certainty rating that YL gave in response to blank trials that did not contain a stimulus, indicating that he was likely hallucinating visual motion. Where the bar reflects the mean of several conditions, the error bars correspond to the standard deviation

Psychophysical testing confirmed that YL’s ability to consciously perceive moving stimuli and accurately discriminate their direction of motion is very much dependent on the spatial frequency of the stimuli. In fact, his ability to discriminate was very good with low-frequency checkerboards (93% accuracy, *p* < 0.001, data pooled from all low-frequency trials) but at chance for high-frequency ones (52% accuracy, *p* = 0.286, data pooled from all high-frequency trials). The accuracy and certainty scores per condition are reported in Table S1 and visualised in Fig. 3.

YL’s certainty ratings in this experiment were interesting. As expected, and in line with his performance, he reported high certainty (67% ± 19%) for low-frequency trials, and lower certainty (29% ± 5%) for high-frequency ones. However, his certainty ratings for the blank trials, during which there was no visual stimulation, were remarkably high (60% ± 38%) and comparable to his ratings for low-frequency trials (*t*(130) = 0.82, *p* = 0.416, *n.s.*); they were in fact much higher than his responses to high-frequency stimuli (*t*(130) = 3.89, *p* < 0.001). This suggested that, when a stimulus is expected but not presented, YL could be hallucinating visual motion. To explore this further, we presented him with a blank screen for 150 s (grey background with a fixation cross) and asked him to verbally respond whenever he detected motion in his blind field. He consistently reported seeing motion, and his experience varied in intensity, e.g., he occasionally described this hallucinated motion by exclaiming “oh, this was a big one!”.

Based on previous reports (ffytche and Zeki 2011; Zeki and ffytche 1998), we predicted that YL’s accuracy and certainty would be tightly coupled, and we tested this hypothesis in several ways. First, we found that the mean accuracy and certainty measures obtained from the eight task conditions were highly correlated: *r* = 0.88, *p* = 0.002, one-tailed. Second, we tested whether certainty values, as predicted by the psychophysical model adopted from Zeki and ffytche (1998) and shown in Fig. 3A, agree with the certainty measures obtained from the task; indeed, this was the case: *r* = 0.88, *p* = 0.002, one-tailed.

We further trained linear models on 75% of the task trials to predict YL’s accuracy and certainty on the remaining 25% of trials. We found that a linear model trained using spatial frequency as a sole predictor of accuracy was as good at predicting whether YL’s response to any trial was correct (log-likelihood = − 76.47) as a model trained using frequency, speed, and contrast as predictors (log-likelihood = − 75.17); this was confirmed by the likelihood ratio test: ratio = 2.61, *p* = 0.260, *n.s*. However, a linear model trained with spatial frequency as a sole predictor of certainty was not as good (log-likelihood = − 50.02) as a model trained with frequency, speed, and contrast (log-likelihood = − 41.42), as confirmed by the likelihood ratio test: ratio = 17.19, *p* = 0.001. The accuracy and certainty scores predicted by the full models for each of the eight task conditions were very strongly corelated with YL’s results, as confirmed by Pearson correlations: *r* = 0.94, *p* < 0.001 for both accuracy and certainty (Fig. S4). More details about the model outputs are available in the supplementary material.

**Fig. 4:**
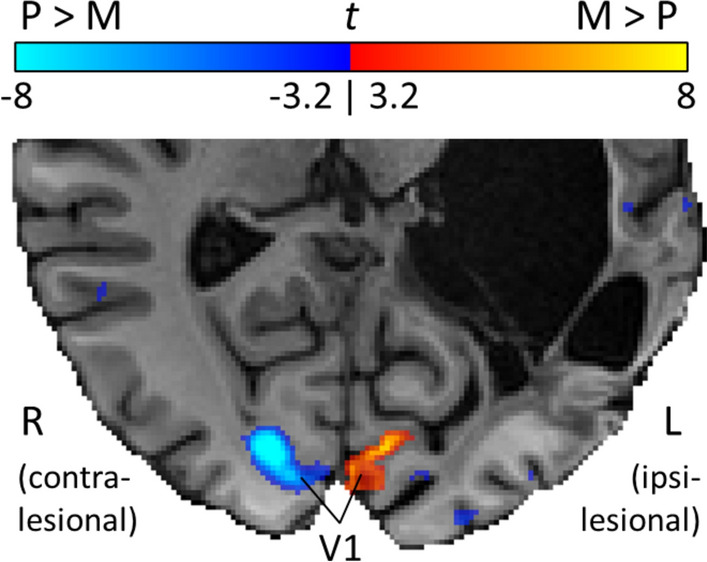
Comparison of neural responses to M and P stimuli in YL’s visual cortex. YL performed an fMRI experiment at 7T in which two visual stimuli were presented bilaterally, one that preferentially engages the magnocellular (M) and the other the parvocellular (P) visual pathway. A direct comparison of the responses to the two stimuli revealed that contralesional V1 activity is much stronger for P compared to M, as expected, but the reverse trend is true for ipsilesional V1, with much stronger responses to M compared to P stimuli

High-resolution fMRI data confirmed the presence of widespread ipsilesional visual cortex activity in response to M- and P-type stimuli following bilateral visual stimulation (Fig. S5). In the contralesional hemisphere, P activity was stronger than M activity within V1 (Fig. 4); this was expected based on previously reported fMRI results (Liu et al. 2006). In contrast, ipsilesional V1 responses to M stimuli were much stronger than those to P stimuli (Fig. 4 and Table S2). This indicates that neural responses in ipsilesional V1 are selectively impaired for P stimuli, which explains why YL is perimetrically blind to static visual input to his right hemifield.

## Discussion

Through our inquiry into patient YL, who has a dense right homonymous hemianopia but can see motion in his blind field, we have demonstrated that the Riddoch syndrome can arise through a disconnection mechanism. In line with YL’s visual impairment and spared motion perception, neural responses to static stimuli in his visual cortex are absent, while visual motion of low-spatial frequency stimuli elicits strong, widespread activity. Yet, tractography showed that his optic radiations, although damaged, still connect his LGN with his visual cortex, including V1. We reconstructed these connections using diffusion MRI data acquired with a very high-diffusion weighting value (*b* = 6000 s/mm^2^), which is mainly sensitive to intra-axonal diffusion (Veraart et al. 2019), rendering the connections anatomically plausible despite their unusual shape due to the tumour’s expansion. So, our findings in patient YL are puzzling: how could V1 receive direct subcortical input, just like prestriate cortex and V5, yet remain unresponsive under certain conditions which should otherwise strongly engage it?

Differential responsiveness of the P and M systems provide a possible explanation for his residual visual abilities. The two systems arise from different populations of retinal ganglion cells; the P system carries information about high-spatial frequencies (high resolution) and requires high-luminance contrast, while the M system is mainly responsive to low-spatial frequencies (low resolution) and fast motion (Kaplan 2014). Therefore, an impaired P system would result in blindness according to static perimetry assessments, while a spared M system would be sufficient for the perception of visual motion. Indeed, psychophysical testing using stimuli of high- and low-spatial frequencies confirmed that YL’s ability to discriminate visual motion direction requires low-frequency inputs. Also, upon further investigation using high-resolution fMRI, we observed that the response properties of his ipsilesional V1 were unusual in that they were much stronger for M stimuli compared with P stimuli, which is the opposite trend of contralesional V1. The detected M signal in V1 is unlikely to be the simple result of feedback from other areas such as V5 because, if this were the case, one would also expect to measure, in V1, strong feedback signals for P inputs from areas such as V2. This response in V1 is also unlikely to be a result of cross-hemispheric feedback during bilateral visual stimulation because, in the first experiment, we observed strong signal changes in ipsilesional visual cortex in response to unilateral visual stimulation of the blind field. We therefore propose that in patient YL thalamic P input to V1 is compromised.

A possible explanation for this differential functional response could be that that M system is more resilient to injury than the P system. Anatomically, M neurons have larger axonal diameters and thicker myelin sheaths, and they can be preferentially spared in the pre-geniculate optic pathway in autoimmune diseases like multiple sclerosis (Evangelou et al. 2001). Alternatively, the M and P systems could be travelling in separate compartments of the optic radiations, e.g., the P compartment may be more lateral to the M compartment, but we could not find evidence for such a clear separation in the literature. Interestingly, Zappia et al. (1971) wrote about two patients who exhibited symptoms of the Riddoch phenomenon and attributed their symptoms to non-cortical origins elsewhere in the visual pathway, such as the optic nerve and optic radiations, though without imaging data to rule out cortical involvement. The weak responses to P stimuli in ipsilesional V1 could be caused by a more localised injury to fibres of the optic radiations that project to V1 but not to other visual areas. This is difficult to assess with imaging, especially given the very narrow anatomical passage that these fibres cross in the compressed white matter of the temporal lobe in YL’s brain.

Alternatively, V1 could be particularly susceptible to any perturbation of the P system. V1 is the largest recipient of LGN input and the largest cortical distributor of visual signals to extrastriate visual cortex (Felleman and Van Essen 1991; Zeki 2015); the processing that occurs within V1 prior to its communication with these areas may be strongly dependent on the quality of thalamic input, which is compromised in YL’s brain. This dependence may be more important for the P system, which is used to extract fine stimulus features such as contours and colour, whereas the M system may be more resilient to such disturbances as it is mainly interested in coarser features of the visual stimulus. Of course, these propositions remain speculative and are difficult to directly confirm with imaging data.

One interesting finding is that YL has a high tendency to report seeing moving stimuli and is certain of correctly discriminating their direction of motion even when none are presented. In fact, YL’s mean certainty score for blank trials (60%) is comparable to his score for low-frequency stimuli (67%), and much higher than that for high frequency ones (29%). So, his certainty responses generally follow the psychophysical model in Fig. 3, in which performance and certainty are tightly linked, but strongly deviate from the model only in the absence of visual stimulation. In the latter case, his high certainty reports suggest that these trials are accompanied by visual hallucinations and are an example of *gnosanopsia*, or awareness without discrimination (Beyh et al. 2023; Zeki and ffytche 1998). Given the partial deafferentation of YL’s visual cortex, we draw a link between his hallucinations and those described in the Charles Bonnet syndrome, which can arise following a mere reduction of visual input that leads to increased cortical excitability (Boroojerdi et al. 2000; Braun et al. 2003; Burke 2002; ffytche, 2005). In YL’s case, his lower confidence on high frequency trials can be explained by the fact that they are accompanied by a visual input, which can regulate visual cortex activity even if YL cannot use this information to perform the task.

In conclusion, we show that a partial disconnection of the optic radiations can lead to a selective loss of visual function where a patient can retain the ability to consciously perceive visual motion despite being blind to static visual stimuli. A differential resilience to injury in the M and P systems may lie at the origin of this anatomical variant of the Riddoch syndrome, in which V1 responses to P stimuli may be selectively impaired after injury to the optic radiations. Therefore, our results raise important questions about the mechanisms that make the M and P systems differentially susceptible to such damage.

### Supplementary Information

Below is the link to the electronic supplementary material.

Supplementary file1 (PDF 1760 KB)

## Data Availability

The data of this study are available upon request from the corresponding author.

## References

[CR1] Ajina S, Kennard C, Rees G, Bridge H (2015). Motion area V5/MT+ response to global motion in the absence of V1 resembles early visual cortex. Brain.

[CR2] Ajina S, Pestilli F, Rokem A, Kennard C, Bridge H (2015). Human blindsight is mediated by an intact geniculo-extrastriate pathway. Elife.

[CR3] Andersson JLR, Graham MS, Drobnjak I, Zhang H, Campbell J (2018). Susceptibility-induced distortion that varies due to motion: correction in diffusion MR without acquiring additional data. Neuroimage.

[CR4] Andersson JLR, Graham MS, Zsoldos E, Sotiropoulos SN (2016). Incorporating outlier detection and replacement into a non-parametric framework for movement and distortion correction of diffusion MR images. Neuroimage.

[CR5] Andersson JLR, Skare S, Ashburner J (2003). How to correct susceptibility distortions in spin-echo echo-planar images: Application to diffusion tensor imaging. Neuroimage.

[CR6] Andersson JLR, Sotiropoulos SN (2016). An integrated approach to correction for off-resonance effects and subject movement in diffusion MR imaging. Neuroimage.

[CR7] Arcaro MJ, Thaler L, Quinlan DJ, Monaco S, Khan S, Valyear KF, Goebel R, Dutton GN, Goodale MA, Kastner S, Culham JC (2019). Psychophysical and neuroimaging responses to moving stimuli in a patient with the Riddoch phenomenon due to bilateral visual cortex lesions. Neuropsychologia.

[CR8] Beckers G, Zeki S (1995). The consequences of inactivating areas V1 and V5 on visual motion perception. Brain.

[CR9] Benevento LA, Rezak M (1976). The cortical projections of the inferior pulvinar and adjacent lateral pulvinar in the rhesus monkey (Macaca mulatta): an autoradiographic study. Brain Res.

[CR10] Beyh A, Rasche SE, Leff A, Ffytche D, Zeki S (2023). Neural patterns of conscious visual awareness in the Riddoch syndrome. J Neurol.

[CR11] Boroojerdi B, Bushara KO, Corwell B, Immisch I, Battaglia F, Muellbacher W, Cohen LG (2000). Enhanced excitability of the human visual cortex induced by short-term light deprivation. Cereb Cortex.

[CR12] Braun CMJ, Dumont M, Duval J, Hamel-Hébert I, Godbout L (2003). Brain modules of hallucination: an analysis of multiple patients with brain lesions. J Psychiatry Neurosci.

[CR13] Burke W (2002). The neural basis of Charles Bonnet hallucinations: a hypothesis. J Neurol Neurosurg Psychiatry.

[CR14] Cragg BG (1969). The topography of the afferent projections in the circumstriate visual cortex of the monkey studied by the nauta method. Vision Res.

[CR15] Dell’Acqua F, Scifo P, Rizzo G, Catani M, Simmons A, Scotti G, Fazio F (2010). A modified damped Richardson-Lucy algorithm to reduce isotropic background effects in spherical deconvolution. Neuroimage.

[CR16] Dell’Acqua F, Simmons A, Williams SCR, Catani M (2013). Can spherical deconvolution provide more information than fiber orientations? Hindrance modulated orientational anisotropy, a true-tract specific index to characterize white matter diffusion. Hum Brain Mapp.

[CR17] Evangelou N, Konz D, Esiri MM, Smith S, Palace J, Matthews PM (2001). Size-selective neuronal changes in the anterior optic pathways suggest a differential susceptibility to injury in multiple sclerosis. Brain.

[CR18] Felleman DJ, Van Essen DC (1991). Distributed hierarchical processing in the primate cerebral cortex. Cereb Cortex.

[CR19] ffytche DH, Guy CN, Zeki S (1995) The parallel visual motion inputs into areas V1 and V5 of human cerebral cortex. Brain, 118(6): 1375–1394. 10.1093/brain/118.6.1375 10.1093/brain/118.6.1375 8595471

[CR20] ffytche DH (2005) Visual hallucinations and the Charles Bonnet syndrome. Current Psychiatry Reports 7(3): 168–179. 10.1007/S11920-005-0050-3/METRICS 10.1007/s11920-005-0050-3 15935130

[CR21] ffytche, D. H., & Zeki, S. (2011). The primary visual cortex, and feedback to it, are not necessary for conscious vision. Brain.

[CR22] Fries, W. (1981). The projection from the lateral geniculate nucleus to the prestriate cortex of the macaque monkey. Proceedings of the Royal Society of London. Series B. Biological Sciences, 213(1190), 73–80. 10.1098/rspb.1981.0054 10.1098/rspb.1981.0054 6117869

[CR23] Greve DN, Fischl B (2009). Accurate and robust brain image alignment using boundary-based registration. Neuroimage.

[CR24] Holmes G (1918). Disturbances of vision by cerebral loss. Br J Ophthalmol.

[CR25] Jenkinson M, Bannister P, Brady M, Smith S (2002). Improved optimization for the robust and accurate linear registration and motion correction of brain images. Neuroimage.

[CR26] Kaplan E, Werner JS, Chalupa LM (2014). The M, P and K Pathways of the Primate Visual System Revisited. The New Visual Neurosciences.

[CR27] Kellner E, Dhital B, Kiselev VG, Reisert M (2016). Gibbs-ringing artifact removal based on local subvoxel-shifts. Magn Reson Med.

[CR28] Liu CSJ, Bryan RN, Miki A, Woo JH, Liu GT, Elliott MA (2006). Magnocellular and parvocellular visual pathways have different blood oxygen level-dependent signal time courses in human primary visual cortex. Am J Neuroradiol.

[CR29] Lutkenhoff ES, Rosenberg M, Chiang J, Zhang K, Pickard JD, Owen AM, Monti MM (2014). Optimized brain extraction for pathological brains (optiBET). PLoS ONE.

[CR30] Riddoch G (1917). Dissociation of visual perceptions due to occipital injuries, with especial reference to appreciation of movement. Brain.

[CR31] Schmid MC, Mrowka SW, Turchi J, Saunders RC, Wilke M, Peters AJ, Ye FQ, Leopold DA (2010). Blindsight depends on the lateral geniculate nucleus. Nature.

[CR32] Sincich LC, Park KF, Wohlgemuth MJ, Horton JC (2004). Bypassing V1: A direct geniculate input to area MT. Nat Neurosci.

[CR33] Tustison NJ, Avants BB, Cook PA, Zheng Y, Egan A, Yushkevich PA, Gee JC (2010). N4ITK: Improved N3 bias correction. IEEE Trans Med Imaging.

[CR34] Veraart J, Fieremans E, Novikov DS (2016). Diffusion MRI noise mapping using random matrix theory. Magn Reson Med.

[CR35] Veraart J, Fieremans E, Novikov DS (2019). On the scaling behavior of water diffusion in human brain white matter. Neuroimage.

[CR36] Vizioli L, Moeller S, Dowdle L, Akçakaya M, De Martino F, Yacoub E, Uğurbil K (2021). Lowering the thermal noise barrier in functional brain mapping with magnetic resonance imaging. Nat Commun.

[CR37] Yukie M, Iwai E (1981). Direct projection from the dorsal lateral geniculate nucleus to the prestriate cortex in macaque monkeys. J Comp Neurol.

[CR38] Zappia RJ, Enoch JM, Stamper R, Winkelman JZ, Gay AJ (1971). The Riddoch phenomenon revealed in non-occipital lobe lesions. Br J Ophthalmol.

[CR39] Zeki S (2015). A massively asynchronous, parallel brain. Philosophical Transactions of the Royal Society B: Biological Sciences.

[CR40] Zeki S, ffytche, D. H. (1998). The Riddoch syndrome: insights into the neurobiology of conscious vision. Brain.

