# Supplementary material for: A clinico-anatomical dissection of the magnocellular and parvocellular pathways in a patient with the Riddoch syndrome
Source: Brain Struct Funct. 2024 Mar 16;229(4):937–46. doi: 10.1007/s00429-024-02774-8 (PMC11004049; doi:10.1007/s00429-024-02774-8)
Supplement: Supplementary file 1 — Supplementary file1 (PDF 1760 KB) [file 429_2024_2774_MOESM1_ESM.pdf]

A clinico-anatomical dissection of the magnocellular and parvocellular pathways in a patient with the Riddoch syndrome

SUPPLEMENTARY MATERIAL

Ahmad Beyh [1,†] & Samuel E Rasche, [1,†] Alexander Leff, [2] Dominic ffytche, [3] Semir Zeki [1,*]

[1] *Laboratory of Neurobiology, University College London, London, UK*
[2] *UCL Queen Square Institute of Neurology, University College London, London, UK*
[3] *Department of Old Age Psychiatry, Institute of Psychiatry, Psychology and Neuroscience, King's College London, London, UK*
[†] *These authors contributed equally*
[*] *Correspondence to s.zeki@ucl.ac.uk*

This document contains supplemental material related to the main manuscript and contains:

- Images of the stimuli used during the experiments.
- Additional behavioural results from the psychophysics task.
- Additional fMRI results.

STIMULI USED DURING THE EXPERIMENTS

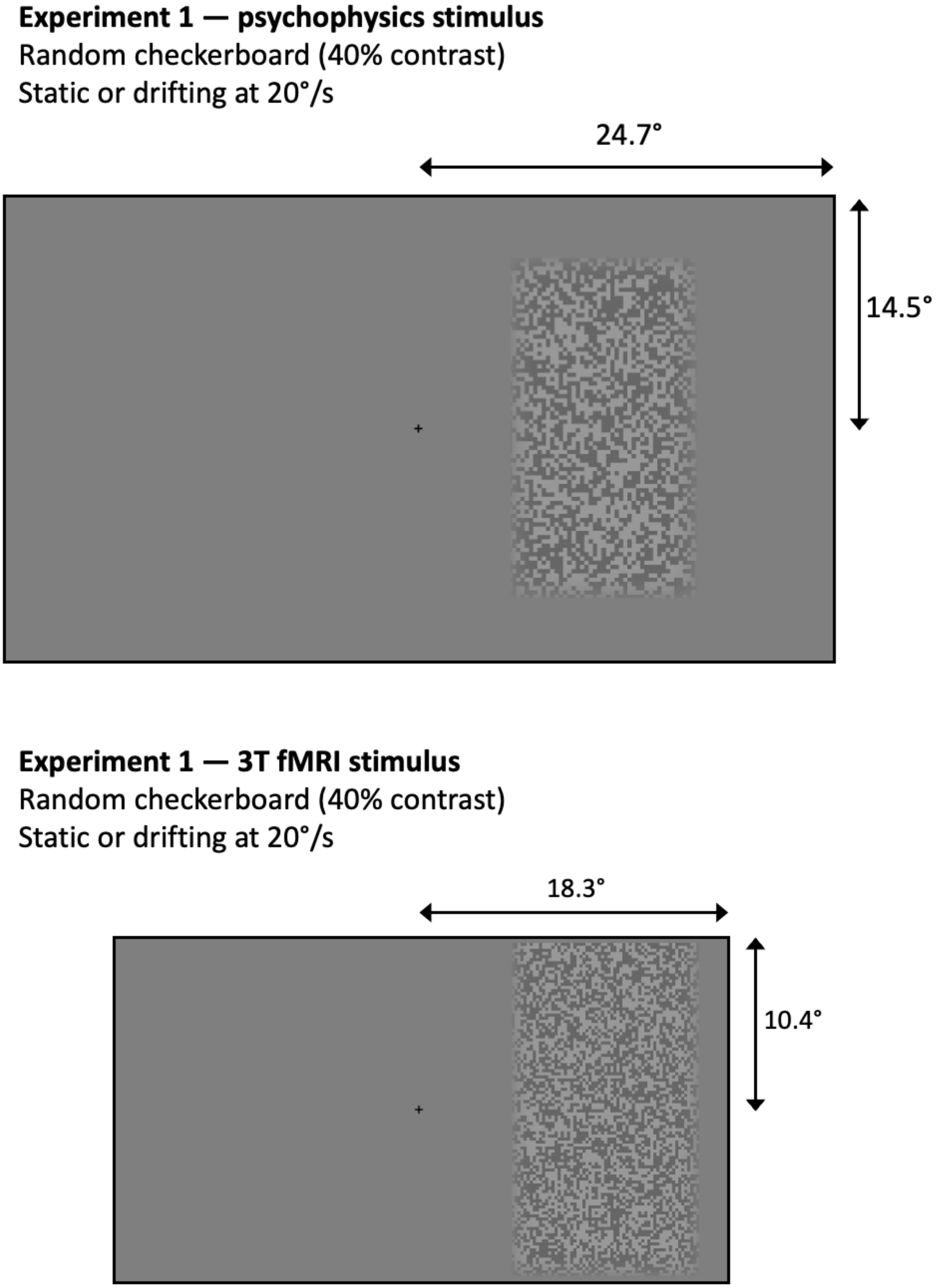

Figure S1. Stimuli used during experiment 1.

Experiment 2 — psychophysics stimulus (P)
Sine wave checkerboard at 1.4 cycles/°
20% or 80% contrast
Drifting at 1°/s or 8°/s

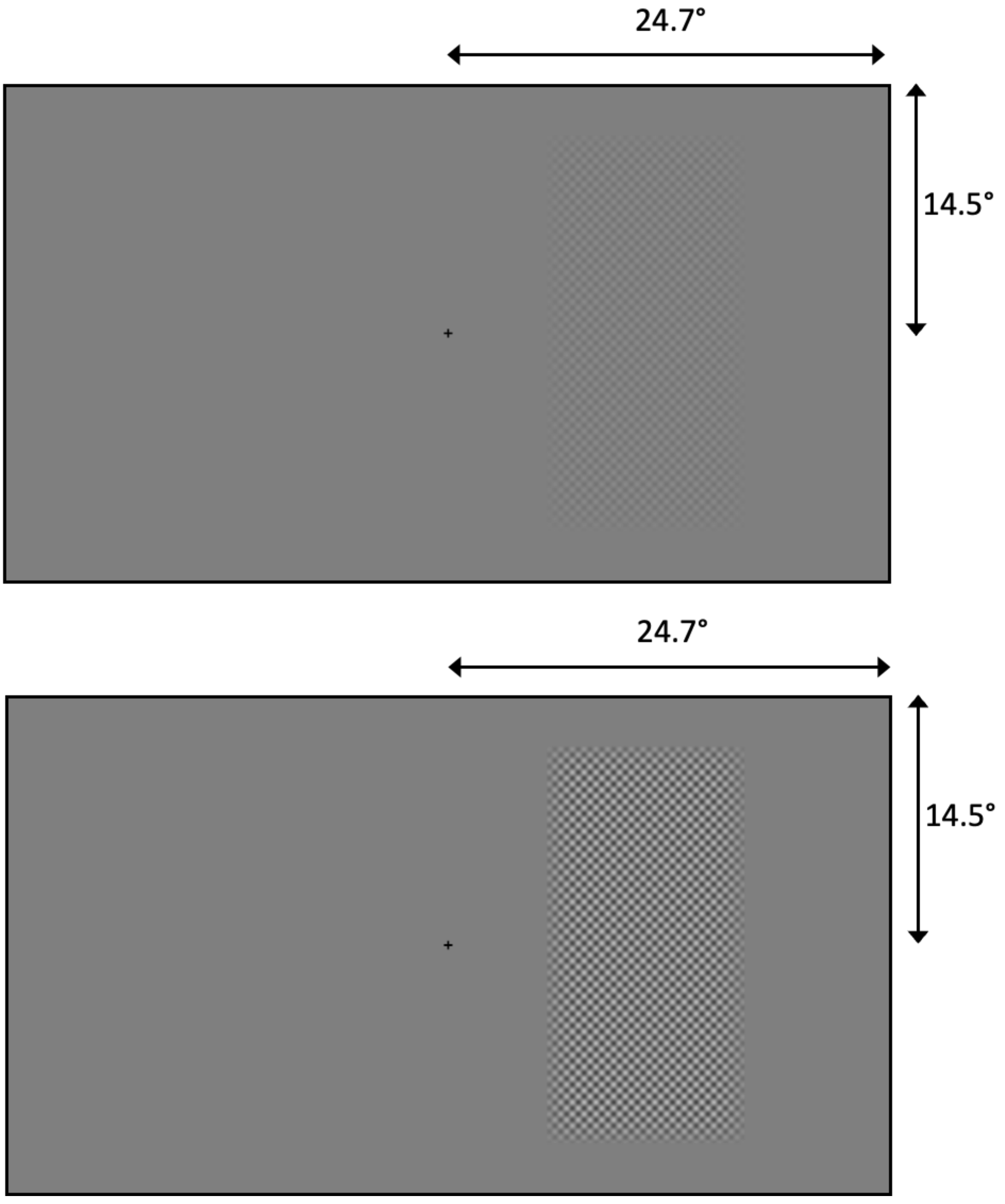

Figure S2A. Stimuli used during experiment 2.

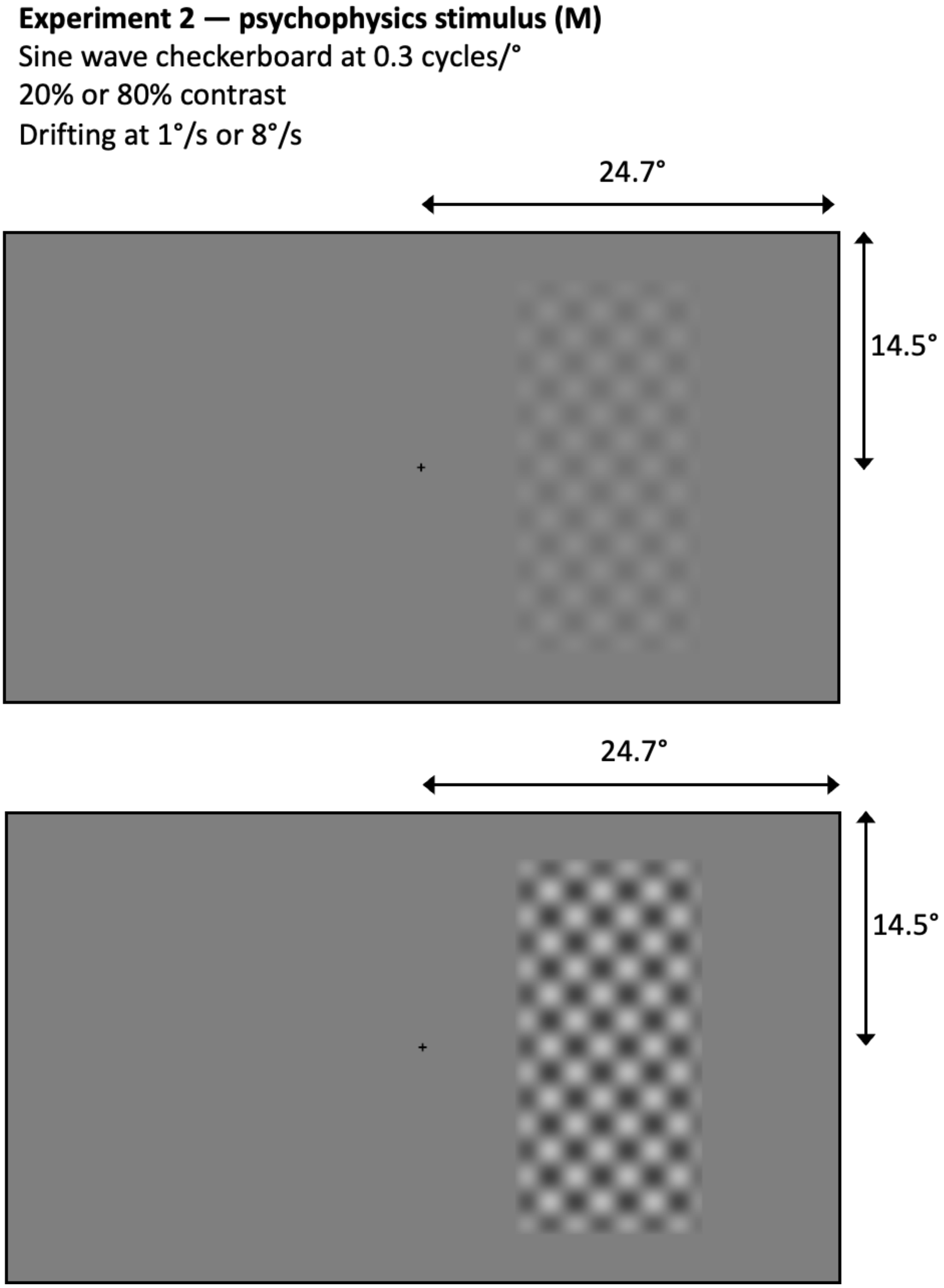

Figure S2B. Stimuli used during experiment 2.

Experiment 2 — 7T fMRI stimulus (P)
Sine wave checkerboard at 1.4 cycles/°
90% contrast
Drifting at 1.5°/s

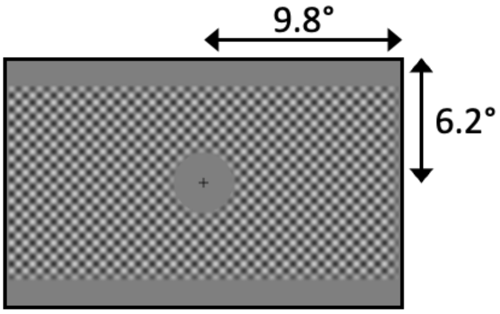

Experiment 2 — 7T fMRI stimulus (M)
Sine wave checkerboard at 0.35 cycles/°
30% contrast
Drifting at 16°/s

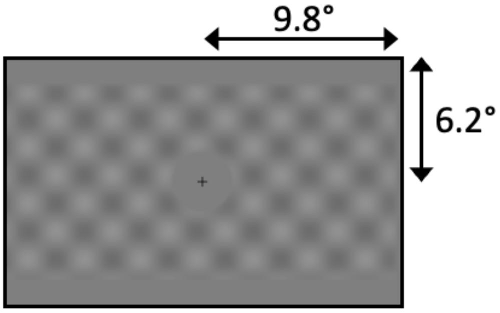

Figure S3. Stimuli used during experiment 2.

SUMMARY OF PSYCHOPHYSICAL RESULTS — EXPERIMENT 2

Table S1. Behavioural results from the second psychophysics session.

	Low spatial frequency (LF)		High spatial frequency (HF)	
	**Low contrast (LC)**	**High contrast (HC)**	**Low contrast (LC)**	**High contrast (HC)**
**Low speed (LS)**	A: 81%*, C: 43%	A: 96%*, C: 75%	A: 50%, C: 27%	A: 50%, C: 30%
**High speed (HS)**	A: 96%*, C: 63%	A: 100%*, C: 88%	A: 65%, C: 25%	A: 44%, C: 36%

Accuracy and certainty scores in visual motion direction discrimination for stimuli varying in contrast, speed, and frequency. *A* represents accuracy in percentages and *C* represents certainty in percentages. For the blank condition, certainty was 60%. *Significantly different from chance performance ($p < .05$) determined from the binomial distribution for 28 trials.

MODELLING OF PSYCHOPHYSICAL RESULTS — EXPERIMENT 2

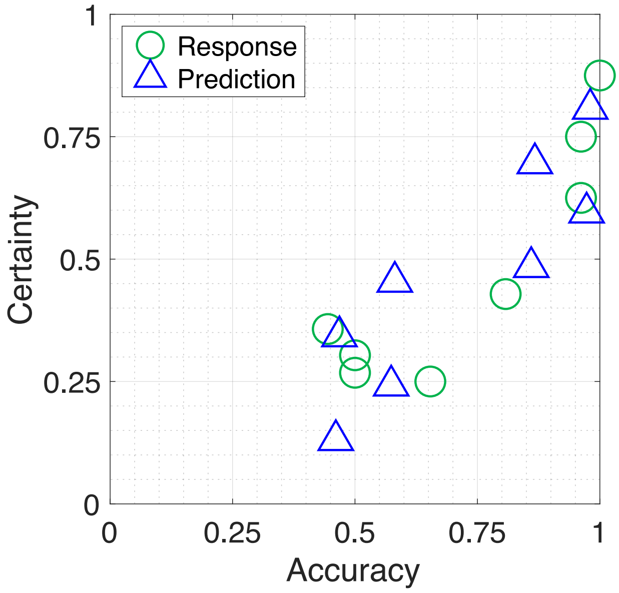

Figure S4. Predicted vs. observed responses during the psychophysics task.
Linear models were trained using 75% of the task trials and used to predict the responses of the remaining 25% of trials. The mean accuracy and certainty of the trials, grouped by task condition, are displayed here. The green circles correspond to YL's actual response data, and the blue triangles correspond to the response data predicted by the models. The models were able to capture the tight relationship between accuracy and certainty. The predictions are based on Model 2 and Model 4 below.

Model 1: Accuracy as a function of spatial frequency

```
Model information:
    Number of observations            158
    Fixed effects coefficients          2
    Random effects coefficients         0
    Covariance parameters               1

Formula:
    Correct ~ 1 + Frequency

Model fit statistics:
    AIC       BIC       LogLikelihood    Deviance
    158.94    168.13    -76.472          152.94

Fixed effects coefficients (95% CIs):
    Name                Estimate    SE          tStat     DF     pValue        Lower       Upper
    {'(Intercept)'}       1.0472    0.057495    18.213    156    1.8751e-40     0.93361      1.1608
    {'Frequency'  }     -0.36824     0.05679   -6.4843    156    1.1157e-09    -0.48042    -0.25606

Random effects covariance parameters (95% CIs):
Group: Error
    Name                  Estimate    Lower      Upper
    {'Res Std'}           0.39261     0.35162    0.43837
```

Model 2: Accuracy as a function of spatial frequency, speed, and contrast

```
Model information:
    Number of observations            158
    Fixed effects coefficients          4
    Random effects coefficients         0
    Covariance parameters               1

Formula:
    Correct ~ 1 + Speed + Contrast + Frequency

Model fit statistics:
    AIC       BIC       LogLikelihood    Deviance
    160.33    175.64    -75.166          150.33

Fixed effects coefficients (95% CIs):
    Name                 Estimate    SE           tStat      DF     pValue        Lower         Upper
    {'(Intercept)'}       0.94849     0.085996     11.029    154    3.2612e-21       0.7786      1.1184
    {'Speed'      }      0.011842    0.0088866     1.3326    154       0.18464   -0.0057133    0.029397
    {'Contrast'   }      0.086808      0.10348    0.83891    154       0.40282     -0.11761     0.29123
    {'Frequency'  }      -0.36442     0.056395     -6.462    154    1.2884e-09     -0.47583    -0.25302

Random effects covariance parameters (95% CIs):
Group: Error
    Name                  Estimate    Lower      Upper
    {'Res Std'}           0.38938     0.34873    0.43477
```

Model 3: Certainty as a function of spatial frequency

```
Model information:
    Number of observations             158
    Fixed effects coefficients           2
    Random effects coefficients          0
    Covariance parameters                1

Formula:
    Certainty ~ 1 + Frequency

Model fit statistics:
    AIC       BIC       LogLikelihood    Deviance
    106.04    115.22    -50.018          100.04

Fixed effects coefficients (95% CIs):
    Name                 Estimate    SE          tStat      DF     pValue        Lower       Upper
    {'(Intercept)'}       0.75316    0.048631     15.487    156     2.332e-33      0.6571     0.84923
    {'Frequency'  }      -0.31646    0.048035    -6.5881    156    6.4732e-10    -0.41134    -0.22157

Random effects covariance parameters (95% CIs):
Group: Error
    Name                   Estimate    Lower      Upper
    {'Res Std'}            0.33208     0.29741    0.37079
```

Model 4: Certainty as a function of spatial frequency, speed, and contrast

```
Model information:
    Number of observations             158
    Fixed effects coefficients           4
    Random effects coefficients          0
    Covariance parameters                1

Formula:
    Certainty ~ 1 + Speed + Contrast + Frequency

Model fit statistics:
    AIC       BIC       LogLikelihood    Deviance
    92.843    108.16    -41.422          82.843

Fixed effects coefficients (95% CIs):
    Name                 Estimate    SE           tStat      DF     pValue         Lower       Upper
    {'(Intercept)'}       0.53274     0.069459     7.6699    154    1.8226e-12      0.39553     0.66995
    {'Speed'      }      0.020442    0.0071776     2.8481    154     0.0049998     0.006263    0.034622
    {'Contrast'   }       0.24921     0.083578     2.9818    154     0.0033323     0.084104     0.41432
    {'Frequency'  }      -0.30987      0.04555    -6.8028    154      2.138e-10    -0.39985    -0.21989

Random effects covariance parameters (95% CIs):
Group: Error
    Name                   Estimate    Lower      Upper
    {'Res Std'}            0.3145      0.28167    0.35116
```

7T FMRI RESULTS

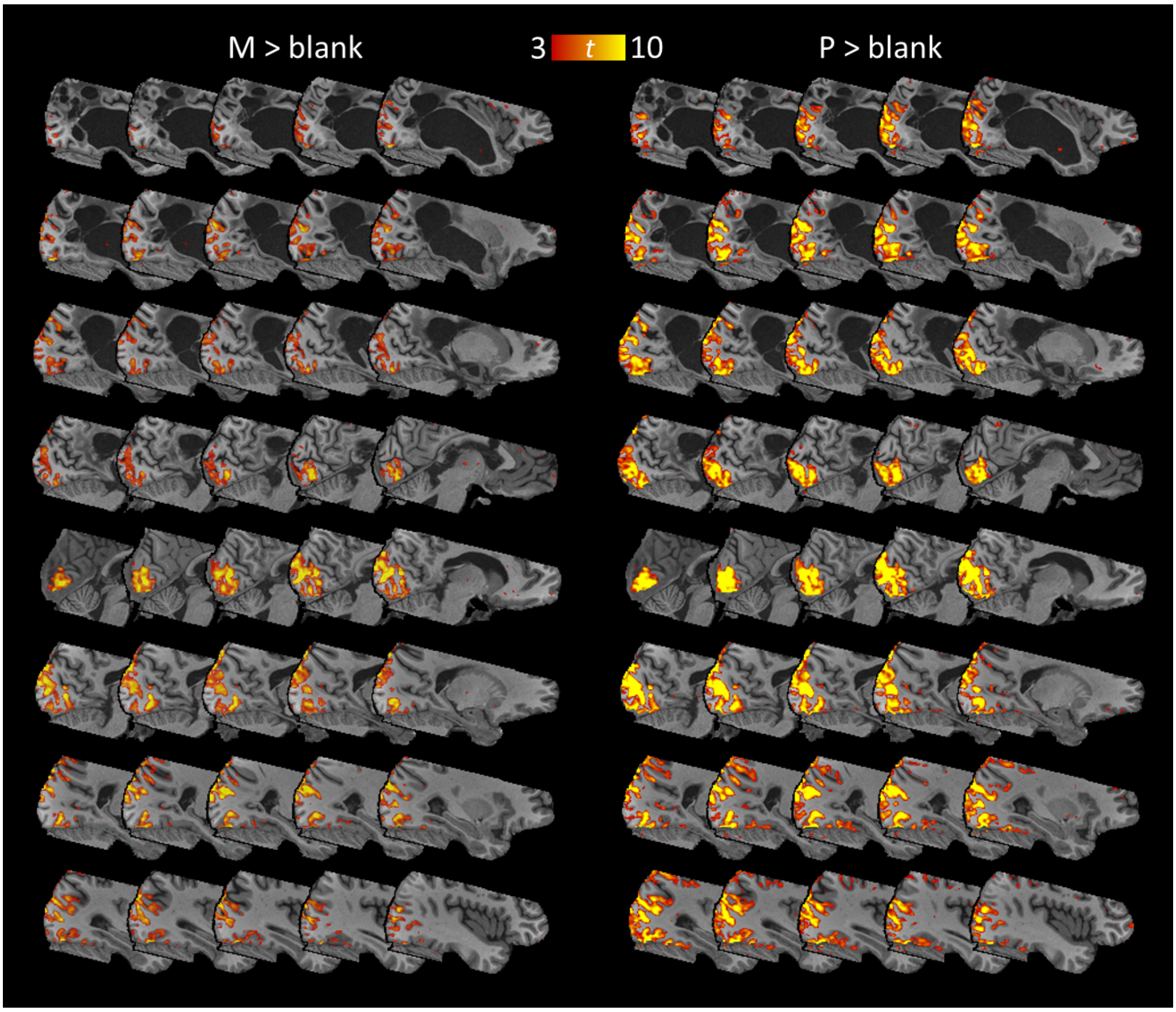

Figure S5. Activity related to the M-type and P-type stimuli in YL's brain.
BOLD activity measured with high-resolution 7T fMRI in relation to a visual motion localiser task. Both M and P were associated with widespread visual cortical activity ($p < .001$).

Table S2. Results of the univariate 7T fMRI analysis.

Cluster and/or region	Voxels	$P_{Clust\text{-}FWE}$	T	Coordinates (mm) x	y	z
M-type stimuli > P-type stimuli						
R V3	61	0.000	7.15	12	-71	0
L V1	2	0.014	5.88	-7	-81	6

Significant activations when comparing the magnocellular-stimulus condition with the parvocellular-stimulus condition. Reported are their corresponding peak t-statistic and coordinates in native space. All results are thresholded at FWE < 0.05.
